# Broad blocking of MDR efflux pumps by acetylshikonin and acetoxyisovalerylshikonin to generate hypersensitive phenotype of malignant carcinoma cells

**DOI:** 10.1038/s41598-018-21710-5

**Published:** 2018-02-22

**Authors:** Seyed Abbas Mirzaei, Somayeh Reiisi, Parmida Ghiasi Tabari, Abolfazl Shekari, Fatemeh Aliakbari, Elaheh Azadfallah, Fatemeh Elahian

**Affiliations:** 1 Cancer Research Center, Shahrekord University of Medical Sciences, Shahrekord, Iran; 2 Department of Genetics, Faculty of Basic Sciences, Shahrekord University, Shahrekord, Iran; 3 Department of Genetics and Molecular Medicine, School of Medicine, Zanjan University of Medical Sciences, Zanjan, Iran; 4 Department of Pharmaceutical Biotechnology, School of Pharmacy, Zanjan University of Medical Sciences, Zanjan, Iran; 5 Cellular and Molecular Research Center, Basic Health Sciences Institute, Shahrekord University of Medical Sciences, Shahrekord, Iran

## Abstract

Cytotoxic activities of acetylshikonin and acetoxyisovalerylshikonin alone and in combination with chemotherapeutic agents against parental and drug resistant cell lines were determined using the MTT assay. Effects of Shikonin derivatives on BCRP, MDR1 and MRP transcript and protein levels were relatively measured. Finally, accumulation and efflux kinetics were conducted. The results revealed cell- and concentration-dependency of the cell cytotoxicity. Acetylshikonin and acetoxyisovalerylshikonin transiently made the mRNA ocean turbulent, but FACS analyses using fluorescent-labeled antibodies showed no significant change in the MDR-protein levels. Functional kinetics revealed significant block of MDR1, BCRP and MRP transporter in the presence of shikonin derivatives. Maximum accumulation fold changes was quantified to be 4.4 and consequently, acetoxyisovalerylshikonin pretreated EPG85.257RDB cells was chemosensitized to daunorubicin tension 3.1-fold. Although, the MDR blockage was reported to follow time- and cell-dependent patterns, MDR1, BCRP and MRP2 responses to the shikonins are concentration-independent. These data suggest uncompetitive transporter blockage behavior of these agents. The results indicated that shikonin derivatives stimulate uptake and reduce efflux of chemotherapeutic agents in the malignant cancer cells, suggesting that chemotherapy in combination with shikonin compounds may be beneficial to cancer cells that overexpress multidrug resistance transporters.

## Introduction

Drug resistance is one of the main challenges in cancer chemotherapy and is influenced by several important factors which include drug potency, cancer cell response to the treatments, tumor microenvironment and heterogeneity of cancer cells^1,2^. Multidrug resistance (MDR) or chemo-resistance represents an event whereby cancer cells exhibit tolerance to a specific chemotherapeutic agent or a class of pharmaceutical drugs^3^. Target-dependent, drug dependent, and drug/target-independent are the most common MDR phenotypes^4,5^. Overexpression of ATP-binding cassette (ABC) transporter superfamilies are the most significant mechanisms underlying MDR phenotype, which consume ATP and efflux either cytotoxic drugs or targeted anticancer agents^6^. The most prevalent ABC superfamily members are including P-glycoprotein (P-gp/ABCB1), multidrug resistance-associated protein (MRP/ABCC), and breast cancer resistance protein (BCRP/ABCG2), which may over-activated or up-regulated in malignant cancers, and develop a distinctive defense contradictory to chemotherapeutics. These pumps clearly reduce the intracellular concentration of frequent endotoxin and exotoxins, therefore causing MDR-phenotype^7,8^.

Recently, many investigators explored herbal bioproducts such as the MDR cell chemosensitizers with least cytotoxicity. Some naturally active ingredients like carotenoid, coumarin, flavonoid, naphthoquinone, stilbenoid, and terpenoid derivatives obtained from plants and fungi have been previously reported as MDR modulators/inhibitors^9,10^. Various studies have revealed that herbal extracts synergistically increased chemotherapy potency, reduced the chemotherapeutics doses, and increased therapeutic window of cytotoxic agents, especially via inverse MDR in cancer cells. These natural MDR-modulators mainly act as antagonists, reverse agonists, transcript down-regulators, protein down-regulators, metabolism modulators, or ATP binding interaction interferers which reduce chemotherapeutics efflux from the cancer cells^10,11^. Naphthazarins and their derivatives have been investigated for a variety of biological functions, such as antibacterial, antimalarial, antifungal and anticancer properties^12,13^.

Shikonin is one of the natural naphthazarins which is the major constituent of red extracts of the roots of *Lithospermum erythrorhizon* and *Alkanna frigida* and has been traditionally used for the treatment of sore throat, injuries, infections the and gastrointestinal disorders^14,15^. Shikonin derivatives including isobutyrylshikonin, acetylshikonin, dimethylacrylshikonin and isovalerylshikonin have also demonstrated selective anticancerous, tyrosine kinase inhibitory, ROS generation, anti-angiogenesis, proteasome inhibitory, anti-inflammatory and anti-glycolysis activities^16,17^. Earlier, shikonin was reported to downregulate MDR1 protein and chemosensitized cells resistance to chemotherapy^18^. There is little information on the modulatory mechanism of alkylated naphthazarin moieties on ABC-transporters, although, higher cancer cytotoxicity via acceleration of apoptotic protein production and triggering of death signaling receptors has been reported for some chemically modified shikonin derivatives^16,19^. Therefore, the present study mainly focused on the restrictive effects of two novel alkylated shikonin derivatives: acetylshikonin and β-acetoxyisovalerylshikonin, on ABC-transporter expression level and function in human cancer cell lines. These results present well-known compounds helpful in the management of malignant multidrug resistant cancers.

## Results

### *In vitro* cytotoxic behaviors of shikonins

Sensitivity of the parental cells and their MDR-resistant lines to shikonin derivatives and standard chemotherapeutic agents were measured. After a 5-day treatment with serial dilutions of chemical agents, a dose-response curve was fitted to the normalized MTT data (Supp. Fig. S1). Table 1 shows the IC_10_ and IC_50_ values for acetylshikonin and acetoxyisovalerylshikonin and IC_50_ data for the chemotherapeutic agents. Acetylshikonin presented higher toxicity than the acetoxyisovalerylshikonin for EPG85.257RDB and MCF7MX cell lines. Primary investigations revealed that shikonin derivatives inhibited cell growth time-, dose- and cell-dependently. In addition, the combination of shikonins with standard chemotherapeutics resulted in a statistical significant improvement of daunorubicin, mitoxantrone or cisplatin toxicity and confirmed synergistic toxicity (P < 0.001, Tables 1 and 2). Acetylshikonin combination therapy mostly showed more prominent toxic effects on both parental and resistant cells. Dose-response curves illustrated significant decreases of viable cell fractions against higher concentrations of the combination therapy (Supp. Fig. S2).

**Table 1:** IC_10_ and IC_50_ values for shikonin derivatives and standard chemotherapeutics agents on the cancer cells.

Compounds	EPG85.257	EPG85.257RDB	MCF7	MCF7MX	A2780	A2780RCIS
Acetylshikonin IC_10_ ± SE^a^	16.03 ± 2.01	0.91 ± 0.17	102.92 ± 7.71	31.92 ± 0.89	22.55 ± 5.10	51.78 ± 5.13
Acetoxyisovalerylshikonin IC_10_ ± SE	0.16 ± 0.11	4.16 ± 0.39	180.14 ± 9.19	108.42 ± 11.25	7.35 ± 1.18	10.32 ± 1.07
Acetylshikonin IC_50_ ± SE	49.51 ± 7.32	18.31 ± 2.76	363.56 ± 27.64	237.60 ± 44.21	73.90 ± 6.19	129.44 ± 11.71
Acetoxyisovalerylshikonin IC_50_ ± SE	1.24 ± 0.80	8.71 ± 2.04	>600	>600	21.44 ± 3.56	30.18 ± 4.35
Daunorubicin IC_50_ ± SE	0.06 ± 0.01	9.06 ± 1.14	ND	ND	0.22 ± 0.01	0.21 ± 0.03
Cisplatin IC_50_ ± SE	ND	ND	ND	ND	1.83 ± 0.19	45.89 ± 1.19
Mitoxantrone IC_50_ ± SE	ND	ND	0.62 ± 0.02	6.46 ± 1.02	ND	ND

^a^Drug concentration (µM) necessary for 10% or 50% inhibition of cell growth after 5 days of drug treatment.

Data display the mean ± standard error of three distinct experiments.

The extrapolated IC_50_ values for acetoxyisovalerylshikonin for MCF7 and MCF7MX cells are 991 and 819 μM, respectively.

ND, not defined.

**Table 2:** Synergistic toxicity of standard chemotherapeutics agent on the cancer cells cotreated either with acetylshikonin or acetoxyisovalerylshikonin.

Compounds	EPG85.257	EPG85.257RDB	MCF7	MCF7MX	A2780	A2780RCIS
Daunorubicin SIC_50_ ± SE and acetylshikonin	0.01 ± 0.01	1.85 ± 0.17	ND	ND	0.07 ± 0.02	0.09 ± 0.01
Daunorubicin SIC_50 ± _SE and acetoxyisovalerylshikonin	0.02 ± 0.00	2.88 ± 0.14	ND	ND	0.03 ± 0.01	0.176 ± 0.08
Mitoxantrone SIC_50_ ± SE and acetylshikonin	ND	ND	0.13 ± 0.04	9.24 ± 1.42	ND	ND
Mitoxantrone SIC_5 0_ ± SE and acetoxyisovalerylshikonin	ND	ND	0.92 ± 0.16	8.16 ± 1.32	ND	ND
Cisplatin SIC_50_ ± SE and acetylshikonin	ND	ND	ND	ND	5.23 ± 1.15	117.84 ± 10.81
Cisplatin SIC_50_ ± SE and acetoxyisovalerylshikonin	ND	ND	ND	ND	7.93 ± 1.04	135.83 ± 9.22

^a^Daunorubicin, Mitoxantrone, or cisplatin concentrations (µM) required for 50% inhibition of cell growth after 5 days of drug exposure.

Data represent the mean ± standard error of three distinct tests.

ND, not defined.

### MDR pump transcripts level and relative protein quantification

**Figure 1:**
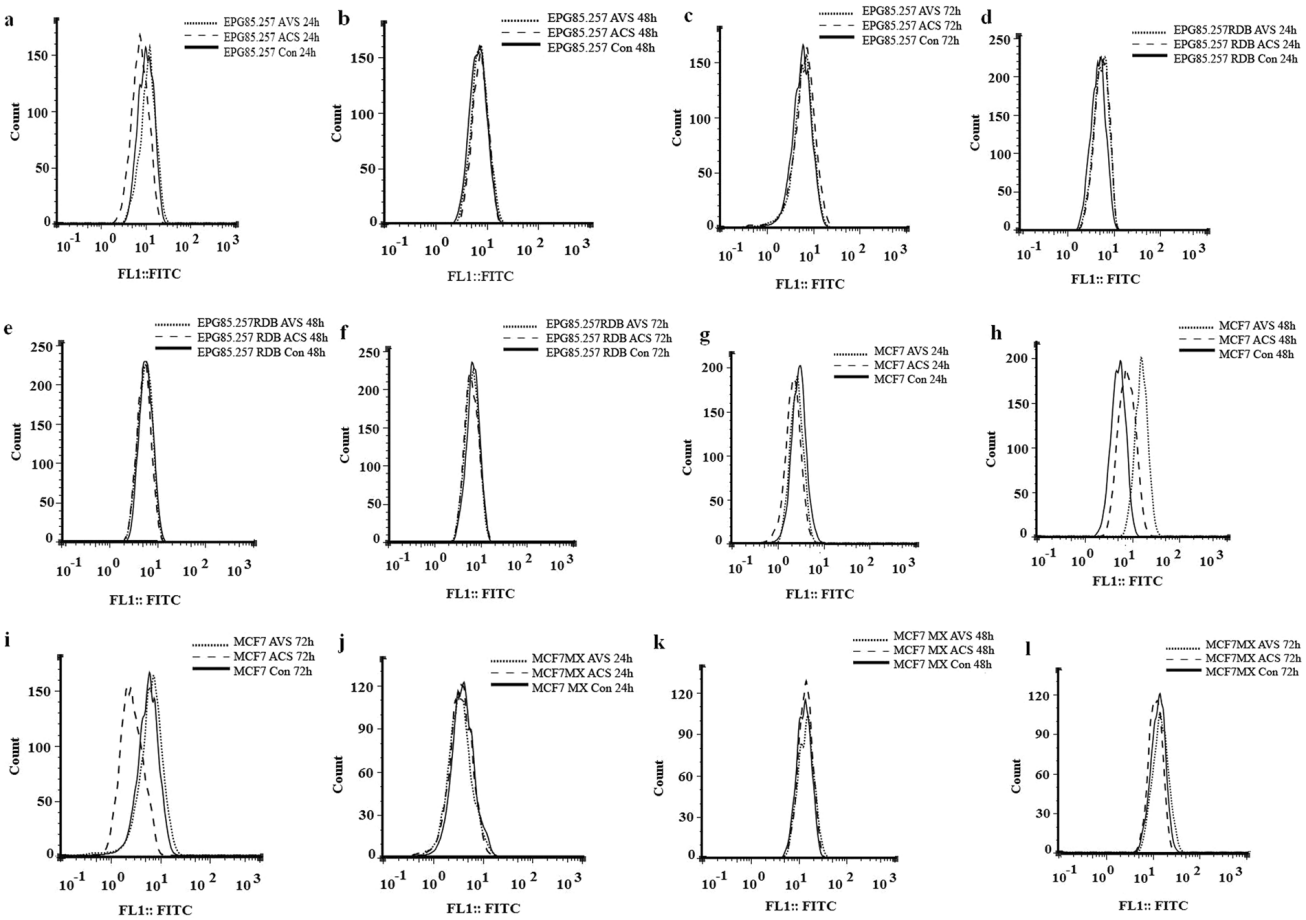
Relative quantification of MDR proteins using flow cytometer. MDR1 and BCRP protein levels of EPG85.257 and MCF7 parental and their resistant counterparts were quantified in the presence of acetylshikonin and acetoxyisovalerylshikonin. ACS, acetylshikonin; AVS, acetoxyisovalerylshikonin; Con, control.

Relative BCRP, MDR1 and MRP transcript and protein quantities were measured using real time PCR and flow cytometer instruments according to the validated standard protocols. Real time based transcript quantifications was optimized on Pfaffl method using high effective primer pairs (Supp. Table S1). Initial experiments showed that MCF7MX, EPG85.257RDB and A2780RCIS cells express BCRP, MDR1 and MRP2 approximately 800-, 310- and 13-fold more than their corresponding parents, respectively. Totally, MDR1 and MRP1 transcripts were up-regulated in the resistant cells time-dependently and BCRP and MRP2 represented down-regulation patterns over time in these cells after shikonin treatments; however, this could not be generalized to all cells, treatments or times (Supp. Fig. S3). Parent cells generally follow the same expression patterns after treatments just like their resistant counterparts, even though they do not express sophisticated MDR pumps (Supp. Fig. S3). Relative BCRP protein quantifications showed up-regulation after 48 h of acetoxyisovalerylshikonin treatment (P < 0.05) and down-regulation after 72 h of acetylshikonin treatment (P < 0.05) in MCF7 cells; however, the resistant EPG85.257RDB and MCF7MX efflux protein fluctuations are statistically insignificant (P > 0.05) over a 3-day of shikonin exposure (Fig. 1).

### Accumulation and efflux kinetic

**Figure 2:**
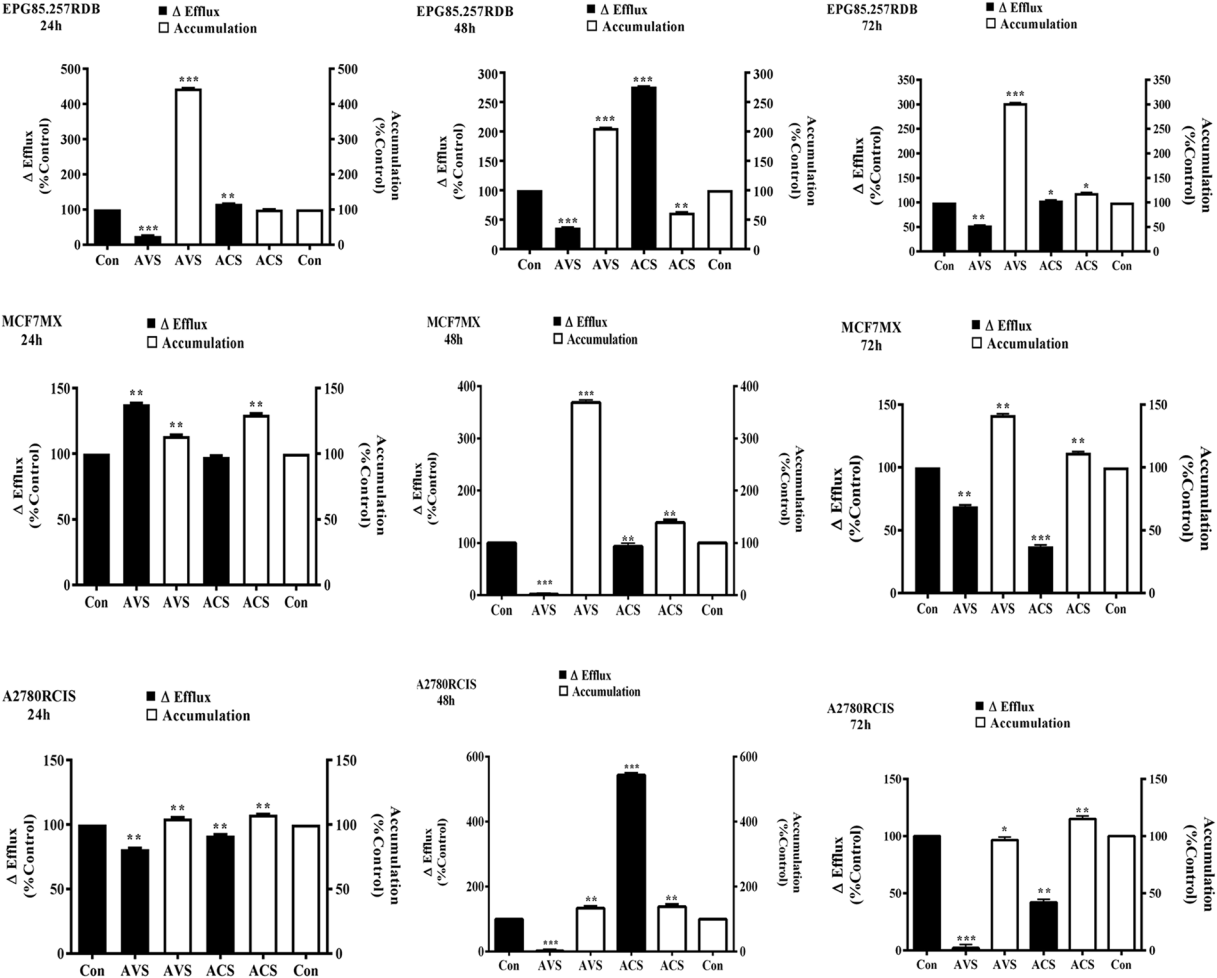
Accumulation and efflux patterns of daunorubicin and mitoxantrone in MDR-resistant cells. Cells were incubated with IC_10_ values of acetylshikonin and acetoxyisovalerylshikonin for 24, 48 and 72 h and then treated with IC_50_ concentration of daunorubicin or mitoxantrone in the presence or absence of a specific inhibitor (indomethacin for MRP2 pumps, verapamil for MDR1 pumps or novobiocin for BCRP pumps). Average fluorescent intensities were recorded. Efflux and accumulation were calculated from corresponding equations. The symbols (***), (**)and (*) represent pump activity differences between shikonin treated and untreated cells as P < 0.001, P < 0.05 and P < 0.01, respectively. ACS, acetylshikonin; AVS, acetoxyisovalerylshikonin; Con, control.

Effects of naphthazarin on the chemotherapeutics accumulation and efflux in the sensitive and resistant cell lines were flow cytometric evaluated. BCRP, MDR1 and MRP2 pump activity was significantly reduced in the acetylshikonin and acetoxyisovalerylshikonin treated MCF7/MX, EPG85.257/RDB and A2780/RCIS cells, respectively (Fig. 2, Supp. Figs S4 and S5). Inconsequence, the shikonins led to a time-dependent accumulation of mitoxantrone and daunorubicin in the respective cells during the incubation periods (P < 0.001). Maximum daunorubicin and mitoxantrone accumulation was detected after 24 and 48 h of acetoxyisovalerylshikonin treatment of EPG85.257RDB and MCF7MX, respectively (P < 0.001). Although, both treatments showed a dynamic reduction in pumps efflux activity, acetoxyisovalerylshikonin resulted in more potent inhibitory activity (P < 0.001). Cells accumulation and efflux pattern of acetylshikonin treated EPG85.257RDB returned to the control levels after 72 h of drug exposure. MRP2 pump activity in A2780RCIS cells was less affected by both treatments in comparison with the other resistant cells (Fig. 2, Supp. Fig. S5).

## Discussion

The main reason for numerous types of chemotherapy failure is overexpressing of MDR pumps in cancer cells^20^. Such cells frequently present increased ATP-dependent efflux pumps feature and demonstrate prominent ability of increased efflux and reduced influx^21^. Numerous herbal-derived extracts and pure constituents are known to increase the chemotherapeutic effects of anticancer agents by modulating the ABC-transporters’ transcription, translation, and activity as well as regulating cell survival, apoptotic signaling pathways, drug distribution, and cellular metabolisms which make them less toxic and promising candidates for targeting MDR resistance^22–24^.

It has been confirmed in the literature that shikonin derivatives exhibit antineoplastic properties mainly by cancer cell growth inhibition, apoptotic pathway induction, DNA topoisomerase interference, antimitogenic action, angiogenesis reduction, and proteasome inhibition^25–27^. Alkylated shikonin derivatives on C2 and C7 showed stronger cytotoxicity on cancer cells rather than normal cells (Supp. Fig. S6). Reports showed that introduction of oxygen-containing alkyls or cycloalkyls on C2 position enhanced the cytotoxicity effects against multiple drug resistant cancer cells^18,28–30^. Table 1 shows results consistent with previous studies and confirms that acetylshikonin and acetoxyisovalerylshikonin with higher levels of oxygen atoms exert remarkable toxic effects on both parental and resistant cancer cells. Although, presence of shorter alkyl groups (2–6 carbons) has been reported to have stronger antitumor activity than longer chains (7–20 carbons) in few researches^31,32^; such patterns could not be extended to A2780/RCIS cells in the present study and Table 1 showed that shikonin toxicity is most likely cell specific. Table 2 presents the interaction of the new derivatives with normal metabolic pathways of MDR-resistant cells which is overall referred to as “Warburg effect”. The mechanisms that enhance this susceptibility may include direct MDR-pump interference, cellular transcription and translation alteration, alteration of oxidoreductase enzymes, ATPase blockage, interruption of gaining energy in tumor cells through the glycolysis pathway, or enhancement of bioavailability of chemotherapeutics which has not been fully investigated yet^33,34^. Though Tables 1 and 2 shows attracting cytotoxic effects of two shikonin analogs on the multidrug resistant cells, evidences on the association between the structural relationship and desired functions of the derivatives remain unclear.

Preliminary results in this study showed up-regulation of MDR1 and MRP1, and down-regulation of BCRP and MRP2 pumps after naphthazarin treatments in the transcriptional levels as primary adaptation and xenobiotic detoxification responses; however, the translational pump levels remained unchanged, and such inconsistency has been reported previously which could be attributed to post-transcriptional changes, the protein half-life and environmental conditions^35–37^. Besides, flow cytometric analyses showed that BCRP, MDR1 and MRP2 efflux function were significantly disrupted during the 72 h treatments (Fig. 2). Acetoxyisovalerylshikonin led to maximum 4-fold daunorubicin accumulation in EPG85.257RDB cells and the corresponding cell was sensitized to daunorubicin toxicity 3-fold. It can be concluded that increased drug accumulation inside these cells is possibly due to inhibition of pump activity after the treatment and resulted in chemotherapy sensitization. Interestingly, an appropriate balance was observed between efflux decrease and accumulation increase responses in the most resistant cells. Mitoxantrone and daunorubicin accumulation in respective MCF7MX and A2780RCIS were less affected by shikonin derivatives and the chemosensitization to the chemotherapeutics was also less observed. MCF7MX cells presented chemotoleration features for mitoxantrone cytotoxicity during both shikonin treatments even though BCRP pump activity was significantly (P < 0.001) blocked during the experiment time. Such behaviors may be attributed to alteration of metabolism pathways of cancer cells associated with energy consumption, chemotherapeutics metabolism and physicochemical inactivation of the hydroxyl naphthoquinones in this cell^36,38^. Our initial investigations demonstrated that chemosensitization and MDR-associated efflux blockage are less likely dependent on shikonin dosage volumes; such separate activities support the hypothesis that shikonins are uncompetitive pump blockers. This phenomenon may due to shikonin derivatives interference in ATP production, distribution or consumption^36,39^. Moreover, ATP transporters that actively efflux xenobiotics gain their energy in tumor cells through the glycolytic pathway. Maybe, inhibiting glycolysis in these cells may also increase the concentrations of chemotherapeutic agents in the cell^34,40,41^.

## Conclusion

The results of the present study indicate that oxygenated shikonin derivatives have potently chemosensitized MDR1 overexpressing cancer cells to the daunorubicin treatment. ABC-transporter blockage follows time- and cell-dependent patterns but MDR1, BCRP and MRP2 responses to the shikonins are concentration-independent. Data suggest that naphthazarin derivatives may be effective uncompetitive transporter blockers. These findings introduced acetylshikonin and acetoxyisovalerylshikonin as attractive candidates for multidrug resistant cancers therapy in clinical practice. However, further investigations are required to uncover unique mechanism of actions and structural activity relationships underlying their effects on efflux transporter.

## Materials and Methods

### Reagents

The chemical were obtained from commercial resources. MTT, chemotherapeutic agents and MDR pump inhibitors were purchased from Sigma-Aldrich (Sigma-Aldrich, Deisenhofen, Germany). Cell culture media and components were purchased from Gibco (Grand Island, NY, USA). BCRP and MDR1, primary antibodies (mouse monoclonal IgG), goat anti-mouse secondary IgG1 and IgG2a conjugated with FITC, and the appropriate isotype controls were obtained from Abcam (Cambridge, USA). Shikonin analogous were generously provided by Professor Alireza Yazdinezhad (Zanjan University of Medical Sciences). All other compounds and solvents were of analytical grade and obtained from Merck Company (Darmstadt, Germany).

### Cell culture and cell viability assays

A2780RCIS (MRP2 overexpressing human epithelial ovarian cancer), EPG85.257RDB (MDR1 overexpressing human gastric adenocarcinoma), MCF7MX (BCRP overexpressing human epithelial breast cancer), and their parental counterparts were generously provided by Professor Herman Lage (Molecular Pathology Department, University Medicine Berlin). Cells were cultured in RPMI-1640 supplemented with 2 mM L-glutamine, 10% FBS, 100 IU/ml penicillin and 100 μg/ml streptomycin in a pre-equilibrated humidified incubator at 37 °C and 5% CO_2_. Media for multidrug resistant A2780RCIS, EPG85.257RDB and MCF7MX cell lines was also supplemented with 33.21 μM cisplatin, 4.74 μM daunorubicin or 0.10 μM mitoxantrone, respectively. Shikonins cytotoxicity was determined after a 5-day incubation of the treated cells by MTT assay at 570 nm using microplate reader. IC_10_ and IC_50_ values were calculated from the scatter plot of the percentage viability versus drug concentrations and defined as the drug concentrations that reduced the surviving cells in the wells by 10 and 50% as compared to the control, respectively. Combination treatment of the cells with defined IC_10_ values of acetylshikonin and acetoxyisovalerylshikonin in the presence of serially diluted cisplatin, daunorubicin or mitoxantrone was also reported as synergistic cytotoxicity. SIC_50_ value was defined as the chemotherapeutic agent concentration that was co-treated with any of shikonins and reduced the surviving fraction of the cells by 50% when compared with the untreated cells. SIC_50_ was measured from scatter plot of relative cell viability versus any of the chemotherapeutic agent concentrations. All the experiments were accomplished three independent times in pentaplicate^42^.

### Relative quantification of the MDR pump transcripts

Cells were seeded at 3 × 10^5^ cells/well and treated with IC_10_ concentrations of acetylshikonin and acetoxyisovalerylshikonin for 24, 48, and 72 h before RNA extraction. High quality total RNA was isolated (RNeasy Mini Kit, Qiagen) and cDNA was generated (QuantiTect ***Reverse Transcription Kit***, Qiagen) from 1 μg RNA following the manufacturer’s instruction. Quantitative transcript analyses were conducted using the QuantiTect SYBR Green PCR kit (Qiagen, Germany). Real time amplification was set to an initial denaturation step for 10 min at 94 C, followed by 40 cycles of amplification (94 °C for 15 s, annealing temperature for 20 s and 72 °C for 25 min) and finally, a normal melting analysis. BCRP, MDR1, MRP1, MRP2 and β-actin primer sequences and properties are provided in the supplementary materials. All the amplification reactions were performed in triplicate and results were normalized to β-actin, and relative fold changes in the expression were quantified according to Pfaffl method^43^.

### Flow cytometry analyses of relative MDR pump levels

Resistant and parent cancer cells were treated with IC_10_ concentrations of acetylshikonin or acetoxyisovalerylshikonin for 24, 48, and 72 h in 6-well plates. Thereafter, the monolayer cells were trypsinized, harvested and finally fixed with formaldehyde (10% v/v) and cold methanol (90% v/v) for 10 min, respectively. Then, the cells were resuspended in blocking suspension of 10% w/v bovine serum albumin for 1 h at room temperature to minimize nonspecific antibody binding. EPG85.257, A2780, MCF7 resistant and parent cells were independently incubated in 0.01 v/v PBS-diluted primary antibody of either human BCRP, MDR1 or MRP2 supplemented with 2% BSA and 0.01% Tween-20 for 1 h at 4 °C. Subsequently, the cells were incubated in PBS buffer containing 0.01 v/v FITC-conjugated goat anti-mouse secondary immunoglobulin, 2% BSA, and 0.01% Tween-20 for 20 min on ice in the dark. PBS-washing was included twice between all steps. Subsequently, the protein levels were analyzed using a FACSCalibur^TM^ flow cytometer with proper negative controls (autofluorescent, secondary antibody and isotype controls). Intact cells were separated from cellular debris using forward/side scatter parameters. FITC-labeled proteins were excited with the 488 nm laser and the emission fluorescence intensity was recorded using photomultiplier tube with a 530/30 nm band-pass filter (FL1). Finally, data were processed using WinMDI (version 2.8) and FlowJo (version 7.6.1) for 3 independent experiments as compared to the corresponding untreated controls^42,43^.

### Effects of shikonin on chemotherapeutics accumulation and efflux kinetic

Briefly, the MDR-resistant cancerous cells and their parental counterparts were seeded at a density of 5 × 10^5^/well in 6-well plates and incubated with IC_10_ values of acetylshikonin and acetoxyisovalerylshikonin for 24, 48 and 72 h. Then, the cells were harvested and divided into two individual groups. First one was treated with only fluorescent chemotherapeutic substrate (1 μM daunorubicin for MDR1 and MRP or 3 μM mitoxantrone for BCRP efflux pumps); the second one received a corresponding specific pump inhibitor also (10 μM verapamil for MDR1, 100 μM indomethacin for MRP or 200 μM novobiocin for BCRP) for 30 min at room temperature. Cells were harvested, PBS-washed and divided into portions. The first fractions were placed on ice and immediately, FACS analyzed for accumulation kinetics. The second fraction was treated with RPMI-1640 containing 10% FBS either with (for inhibitor-treated cells in the accumulation step) or without (for inhibitor-untreated cells in the accumulation step) pump inhibitor for another 1 h in the dark at room 37 °C. After PBS washing for efflux assay, these cells were subjected to FACS analyses. IC_10_ values of shikonin derivatives were presented in all the steps of kinetic assays. Forward/side scatter gating included the viable single cells of the study and the fluorophores were excited at 488 nm. Daunorubicin and mitoxantrone emission was recorded at FL2 (585 nm) and FL3 (670 nm), respectively. 10^5^ events were recorded for samples and the following equations were used for the mathematical models where, MFI, modulator, sample and control are mean fluorescent intensity, pump inhibitors, shikonin treated cells and untreated cells, respectively. Appropriate negative controls were also included in the study and the experiments were repeated at least three independent times^42,44^.

$$\begin{array}{ccc}{\rm{\Delta }}\text{Efflux} & = & ({{\rm{M}}{\rm{F}}{\rm{I}}}_{(\text{with}\,{\rm{m}}{\rm{o}}{\rm{d}}{\rm{u}}{\rm{l}}{\rm{a}}{\rm{t}}{\rm{o}}{\rm{r}}\,\text{sample})}-{{\rm{M}}{\rm{F}}{\rm{I}}}_{(\text{without}\,{\rm{m}}{\rm{o}}{\rm{d}}{\rm{u}}{\rm{l}}{\rm{a}}{\rm{t}}{\rm{o}}{\rm{r}}\,\text{sample})})\\  &  & \,\div\,({{\rm{M}}{\rm{F}}{\rm{I}}}_{(\text{with}\,{\rm{m}}{\rm{o}}{\rm{d}}{\rm{u}}{\rm{l}}{\rm{a}}{\rm{t}}{\rm{o}}{\rm{r}}\,\text{control})}-{\rm{M}}{\rm{F}}{{\rm{I}}}_{(\text{without}\,{\rm{m}}{\rm{o}}{\rm{d}}{\rm{u}}{\rm{l}}{\rm{a}}{\rm{t}}{\rm{o}}{\rm{r}}\,\text{control})})\\ {\rm{D}}{\rm{r}}{\rm{u}}{\rm{g}}\,{\rm{a}}{\rm{c}}{\rm{c}}{\rm{u}}{\rm{m}}{\rm{u}}{\rm{l}}{\rm{a}}{\rm{t}}{\rm{i}}{\rm{o}}{\rm{n}} & = & {{\rm{M}}{\rm{F}}{\rm{I}}}_{(\text{without}\,{\rm{m}}{\rm{o}}{\rm{d}}{\rm{u}}{\rm{l}}{\rm{a}}{\rm{t}}{\rm{o}}{\rm{r}}\,\text{sample})}\div{{\rm{M}}{\rm{F}}{\rm{I}}}_{(\text{without}\,{\rm{m}}{\rm{o}}{\rm{d}}{\rm{u}}{\rm{l}}{\rm{a}}{\rm{t}}{\rm{o}}{\rm{r}}\,\text{control})}\end{array}$$

### Statistical analyses

All the experiments were carried out in triplicates and means ± standard errors were reported. The obtained results were subjected to statistical analyses using Student’s t-test or one-way ANOVA with SPSS-22 software. Results that showed *P*-values less than 0.05 were considered to be statistically significant.

### Data availability

The datasets generated and/or analyzed during the current study are available on request from the corresponding author.

### Ethical approval

This article does not contain any studies with human participants or animals performed by any of the authors.

## Electronic supplementary material

Supplementary information
