# Supplementary material for: Broad blocking of MDR efflux pumps by acetylshikonin and acetoxyisovalerylshikonin to generate hypersensitive phenotype of malignant carcinoma cells
Source: Sci Rep. 2018 Feb 22;8:3446. doi: 10.1038/s41598-018-21710-5 (PMC5823906; doi:10.1038/s41598-018-21710-5)
Supplement: Supplementary file 1 — Supplementary information [file 41598_2018_21710_MOESM1_ESM.pdf]

Scientific Reports
Supplementary Dataset

Broad blocking of MDR efflux pumps by acetylshikonin and acetoxyisovalerylshikonin to generate hypersensitive phenotype of malignant carcinoma cells

Seyed Abbas Mirzaei [1], Somayeh Reiisi [2], Parmida Ghiasi Tabari [1], Abolfazl Shekari [3], Fatemeh Aliakbari [4], Elaheh Azadfallah [4], and Fatemeh Elahian [5,*]

[1] Cancer Research Center, Shahrekord format University of Medical Sciences, Shahrekord, Iran.

[2] Department of Genetics, Faculty of Basic Sciences, Shahrekord University, Shahrekord, Iran.

[3] Department of Genetics and Molecular Medicine, School of Medicine, Zanjan University of Medical Sciences, Iran.

[4] Department of Pharmaceutical Biotechnology, School of Pharmacy, Zanjan University of Medical Sciences, Iran.

[5] Cellular and Molecular Research Center, Basic Health Sciences Institute, Shahrekord University of Medical Sciences, Shahrekord, Iran.

[*]Author for Correspondence:

F. Elahian

Cellular and Molecular Research Center, Basic Health Sciences Institute, Shahrekord University of Medical Sciences, Shahrekord, Iran.

Tel: (+98) 38 33331471, Fax: (+98) 38 33330709

E-mail: elahian@skums.ac.ir, dr.elahian@yahoo.com

Supplementary Table S1. Primer designing and properties

No.	Gene name	Sense primer	Antisense primer	Annealing temperature	Gene accession number at NCBI
**1**	BCRP	5'–TAT CAA TGG GAT CAT GAA ACC TGG-3'	5'–GCG GTG CTC CAT TTA TCA GAA C-3'	62 °C	NM_004827.2
**2**	MDR1	5'–CAG CTA TTC GAA GAG TGG GC-3'	5'–CCT GAC TCA CCA CAC CAA TG-3'	56 °C	NM_000927.4
**3**	MRP1	5' –AGA GAC AGC TCA GCA GCT CCT-3'	5' –GCC TTG TCA GCC TCC ATC AG-3'	59 °C	NM_004996.3
**4**	MRP2	5'–CTA CTC CAT CAA TGA TAA TCT GAC C-3'	5'–AGG ATG ACA TCA GAA ATA GAG ACC-3'	59 °C	NM_000392.3
**5**	β-actin	5'–TCA TGA AGT GTG ACG TGG ACA TC- 3'	5'–CAG GAG GAG CAA TGA TCT TGA TCT- 3'	59 °C	NM_001101.3

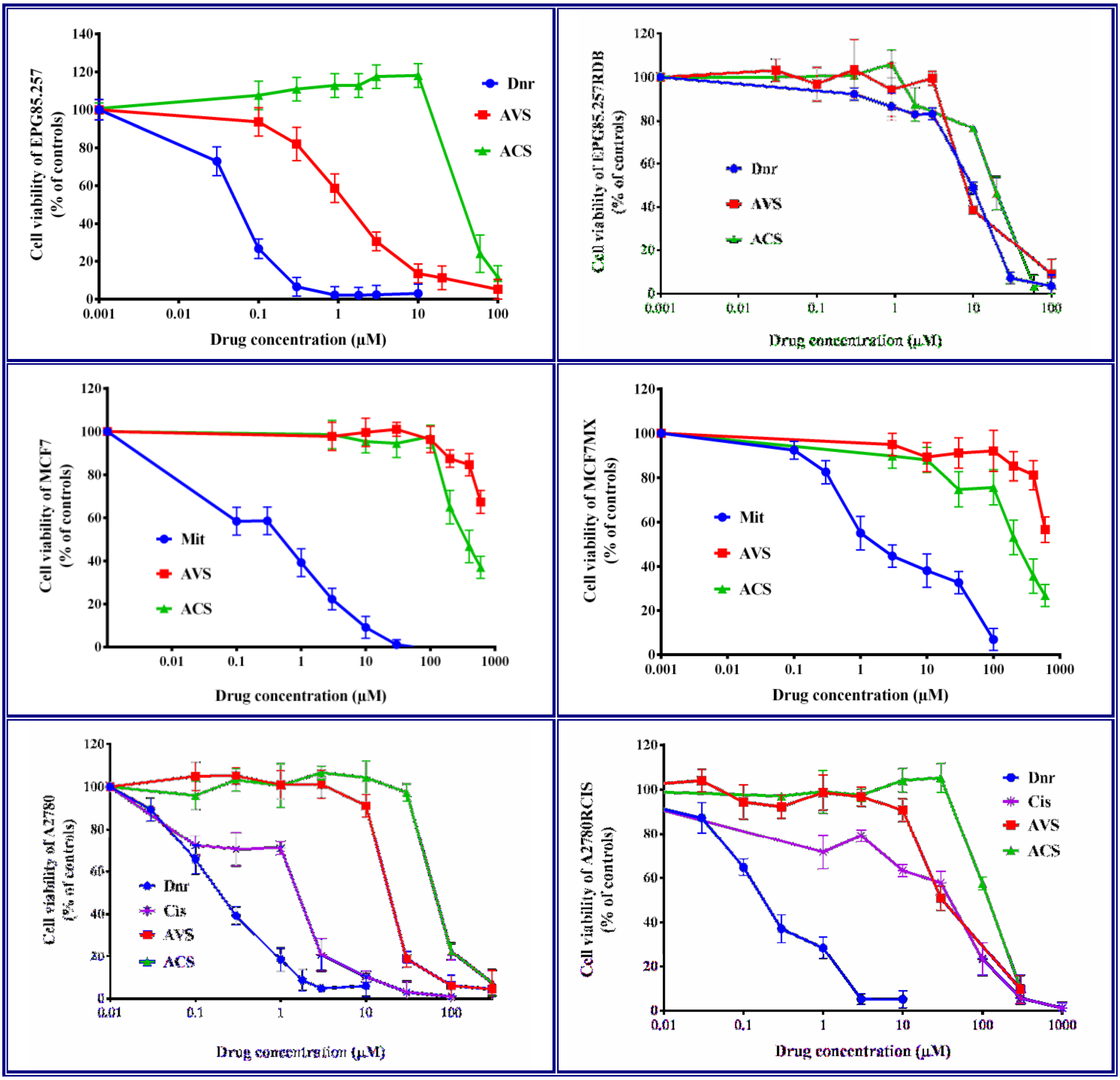

Supplementary Figure S1. Cytotoxic effects of cisplatin, daunorubicin, mitoxantrone, acetylshikonin (ACS), and acetoxyisovalerylshikonin (AVS). Cells were cultured for 5 days with different doses of the agents. Cell viability was evaluated by MTT. The values represent the means of three independent experiments performed in pentaplicate (mean ± SE).

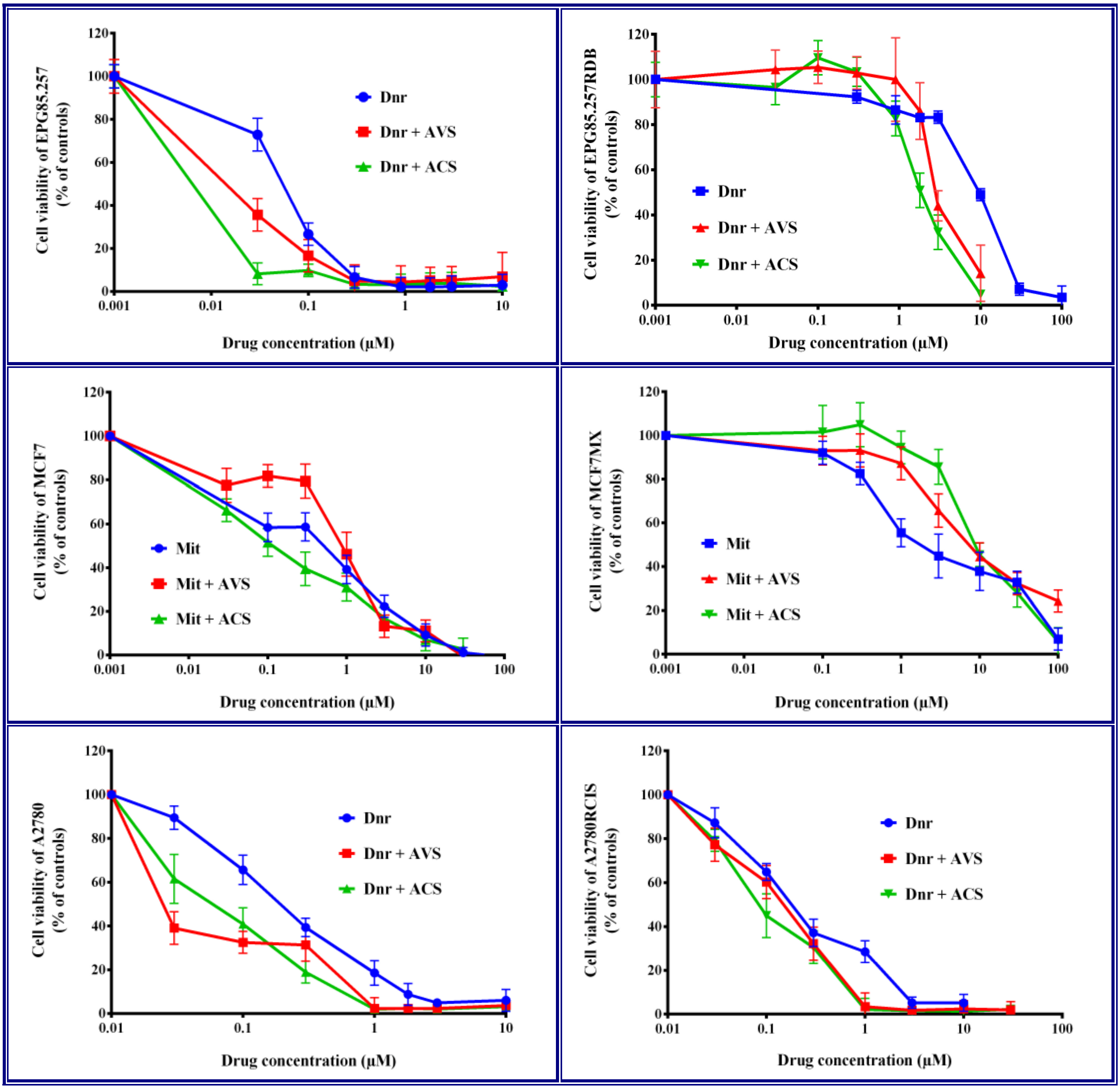
Cell viability of EPG85.257 (% of controls)
Drug concentration (µM)
Dnr
Dnr + AVS
Dnr + ACS
Cell viability of EPG85.257RDB (% of controls)
Drug concentration (µM)
Dnr
Dnr + AVS
Dnr + ACS
Cell viability of MCF7 (% of controls)
Drug concentration (µM)
Mit
Mit + AVS
Mit + ACS
Cell viability of MCF7MX (% of controls)
Drug concentration (µM)
Mit
Mit + AVS
Mit + ACS
Cell viability of A2780 (% of controls)
Drug concentration (µM)
Dnr
Dnr + AVS
Dnr + ACS
Cell viability of A2780RCIS (% of controls)
Drug concentration (µM)
Dnr
Dnr + AVS
Dnr + ACS

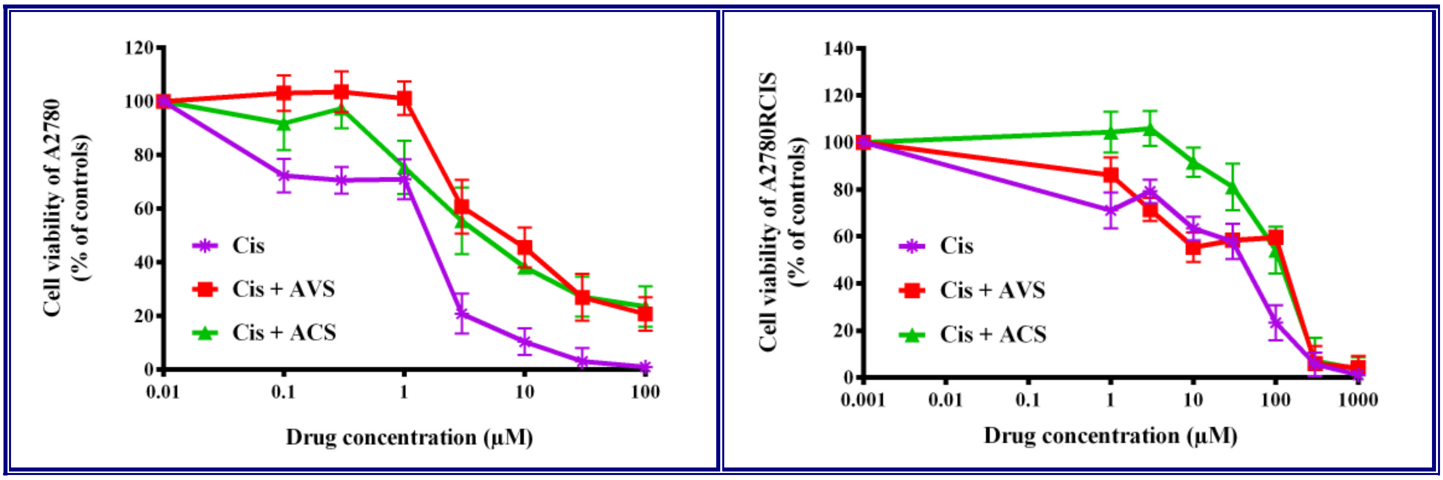

Supplementary Figure S2. Cytotoxicity of cisplatin, daunorubicin, and mitoxantrone either with acetylshikonin (ACS) or acetoxyisovalerylshikonin (AVS). Cells were cultured for 5 days with increasing doses of any chemotherapeutic agents in the presence (IC_{10} values) of each shikonin derivative. The values represent the means of three independent experiments performed in pentaplicate (mean ± SE).

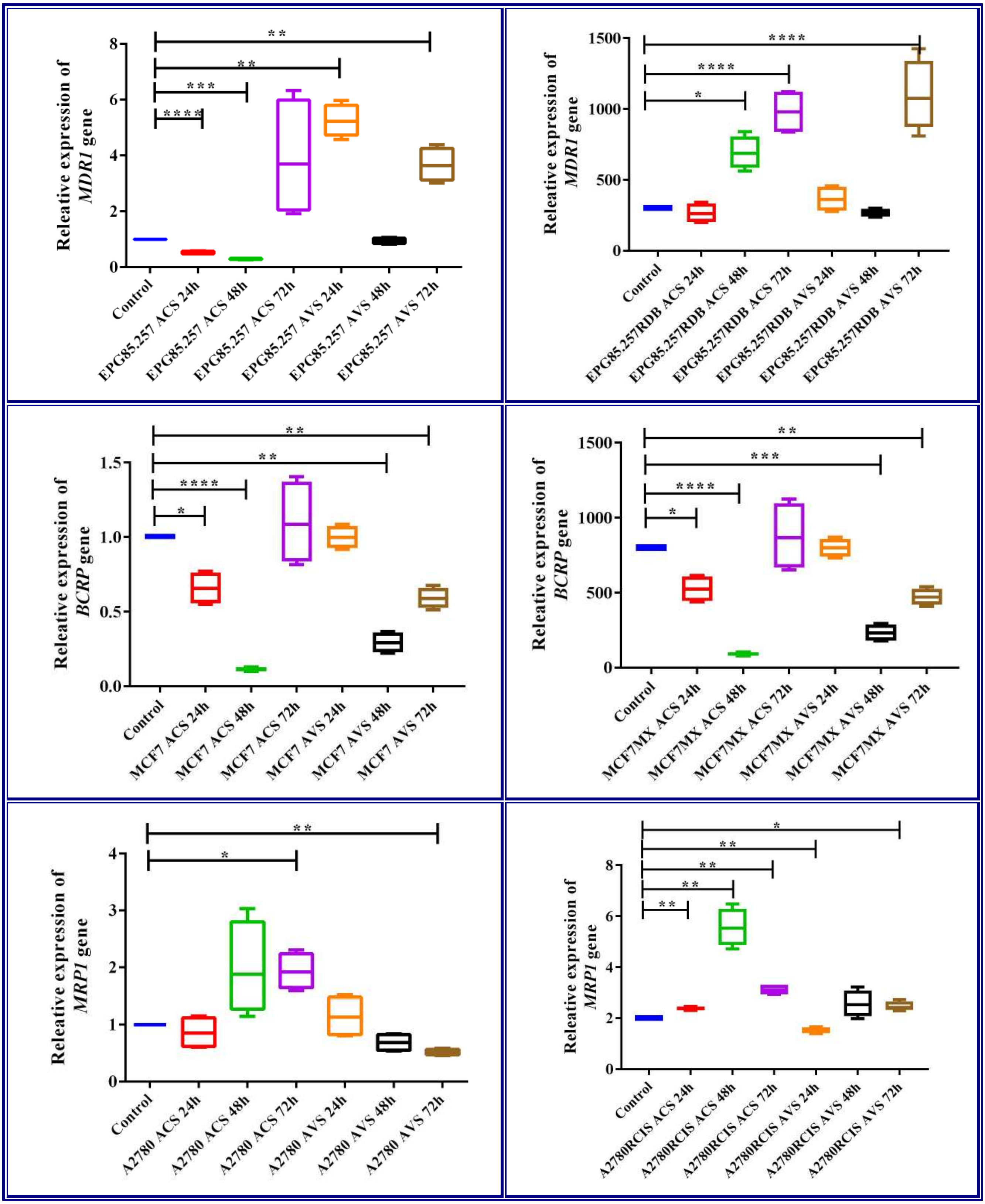
Releative expression of MDR1 gene
0
2
4
6
8
* *
* *
* * *
* * * *
Control
EPG85.257 ACS 24h
EPG85.257 ACS 48h
EPG85.257 ACS 72h
EPG85.257 AVS 24h
EPG85.257 AVS 48h
EPG85.257 AVS 72h
Releative expression of MDR1 gene
0
500
1000
1500
* * * *
* * * *
*
Control
EPG85.257RDB ACS 24h
EPG85.257RDB ACS 48h
EPG85.257RDB ACS 72h
EPG85.257RDB AVS 24h
EPG85.257RDB AVS 48h
EPG85.257RDB AVS 72h
Releative expression of BCRP gene
0.0
0.5
1.0
1.5
* *
* *
* * * *
*
Control
MCF7 ACS 24h
MCF7 ACS 48h
MCF7 ACS 72h
MCF7 AVS 24h
MCF7 AVS 48h
MCF7 AVS 72h
Releative expression of BCRP gene
0
500
1000
1500
* *
* * *
* * * *
*
Control
MCF7MX ACS 24h
MCF7MX ACS 48h
MCF7MX ACS 72h
MCF7MX AVS 24h
MCF7MX AVS 48h
MCF7MX AVS 72h
Releative expression of MRP1 gene
0
1
2
3
4
* *
*
Control
A2780 ACS 24h
A2780 ACS 48h
A2780 ACS 72h
A2780 AVS 24h
A2780 AVS 48h
A2780 AVS 72h
Releative expression of MRP1 gene
0
2
4
6
8
*
* *
* *
* *
* *
Control
A2780RCIS ACS 24h
A2780RCIS ACS 48h
A2780RCIS ACS 72h
A2780RCIS AVS 24h
A2780RCIS AVS 48h
A2780RCIS AVS 72h

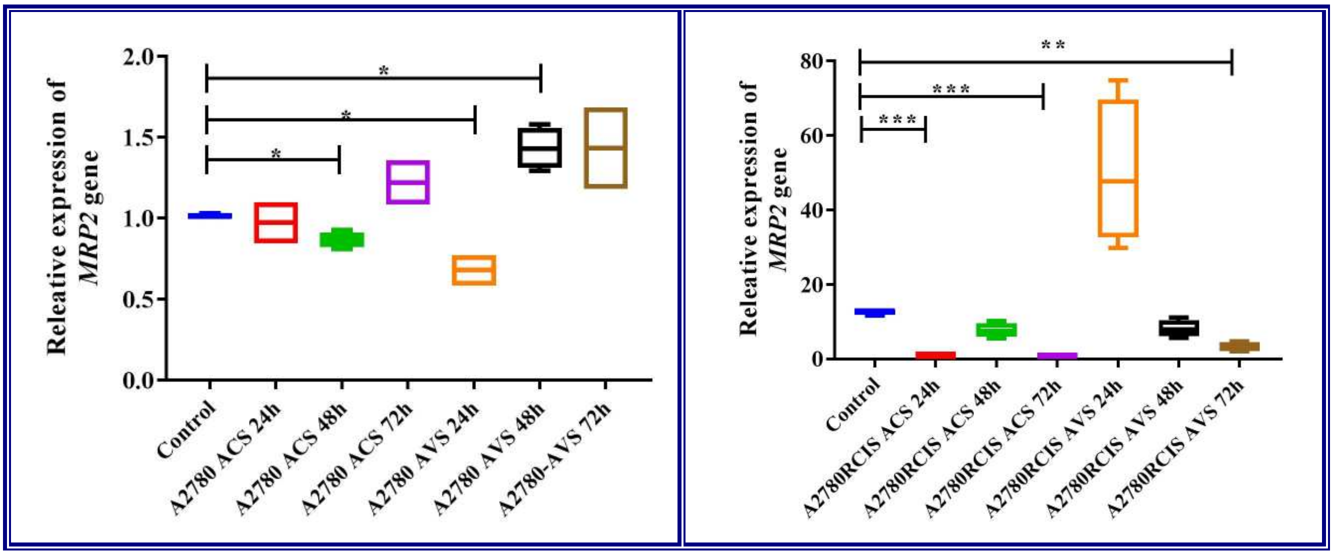

Supplementary Fig. S3. Real-time PCR based quantification of *MDR1, BCRP, MRP1*, and *MRP2* transcripts in EPG85.257RDB, MCF7MX, A2780RCIS and their parental counterparts. Cells were incubated with IC_{10} concentrations of any oxygenated shikonins for 24 h, 48 h, and 72 h. Transcripts levels were measured using the Pfaffl method. The results are reported as mean ± SE of three independent experiments in triplicate. Symbols (∗∗∗∗), (∗∗∗), (∗∗), and (∗) represent the mean differences between parental and resistant cell transcriptions as $P<0.0001$, $P<0.001$, $P<0.01$, and $P<0.05$ using one-way ANOVA, respectively. ACS, acetylshikonin; AVS, acetoxyisovalerylshikonin.

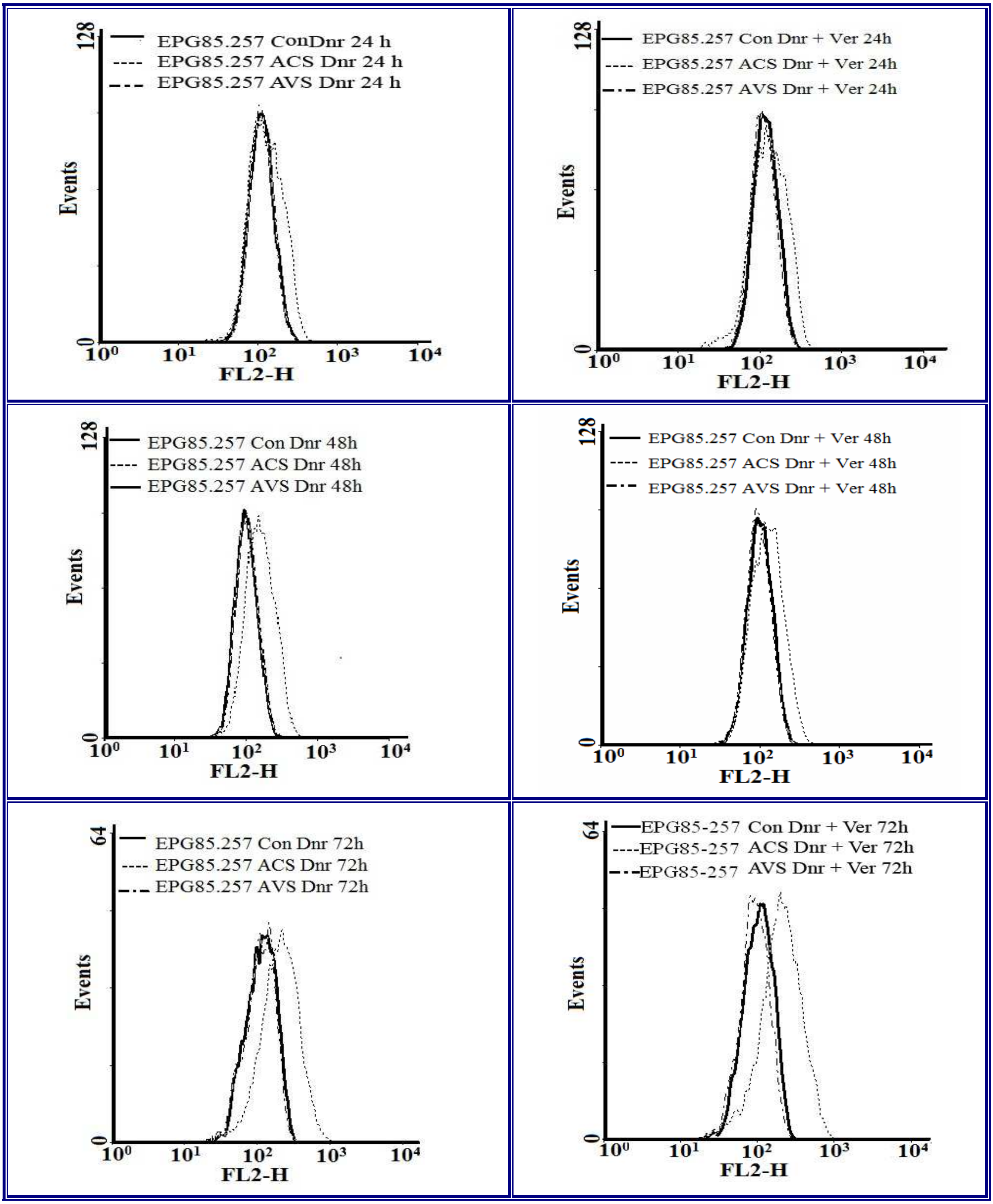
EPG85.257 ConDnr 24 h
EPG85.257 ACS Dnr 24 h
EPG85.257 AVS Dnr 24 h
EPG85.257 Con Dnr + Ver 24h
EPG85.257 ACS Dnr + Ver 24h
EPG85.257 AVS Dnr + Ver 24h
EPG85.257 Con Dnr 48h
EPG85.257 ACS Dnr 48h
EPG85.257 AVS Dnr 48h
EPG85.257 Con Dnr + Ver 48h
EPG85.257 ACS Dnr + Ver 48h
EPG85.257 AVS Dnr + Ver 48h
EPG85.257 Con Dnr 72h
EPG85.257 ACS Dnr 72h
EPG85.257 AVS Dnr 72h
EPG85-257 Con Dnr + Ver 72h
EPG85-257 ACS Dnr + Ver 72h
EPG85-257 AVS Dnr + Ver 72h
Events
128
64
0
FL2-H
10^0
10^1
10^2
10^3
10^4

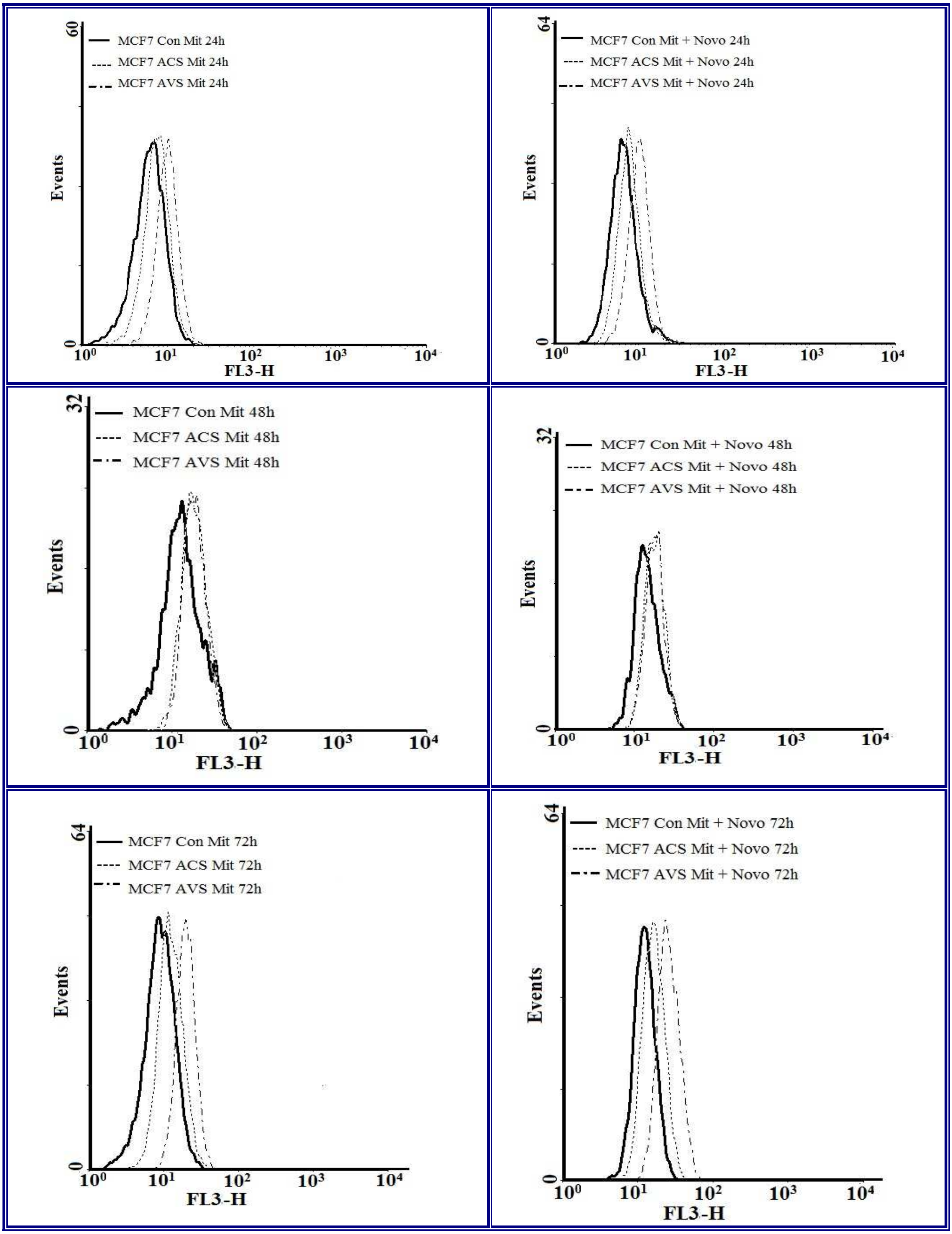
MCF7 Con Mit 24h
MCF7 ACS Mit 24h
MCF7 AVS Mit 24h
MCF7 Con Mit + Novo 24h
MCF7 ACS Mit + Novo 24h
MCF7 AVS Mit + Novo 24h
MCF7 Con Mit 48h
MCF7 ACS Mit 48h
MCF7 AVS Mit 48h
MCF7 Con Mit + Novo 48h
MCF7 ACS Mit + Novo 48h
MCF7 AVS Mit + Novo 48h
MCF7 Con Mit 72h
MCF7 ACS Mit 72h
MCF7 AVS Mit 72h
MCF7 Con Mit + Novo 72h
MCF7 ACS Mit + Novo 72h
MCF7 AVS Mit + Novo 72h
Events
FL3-H

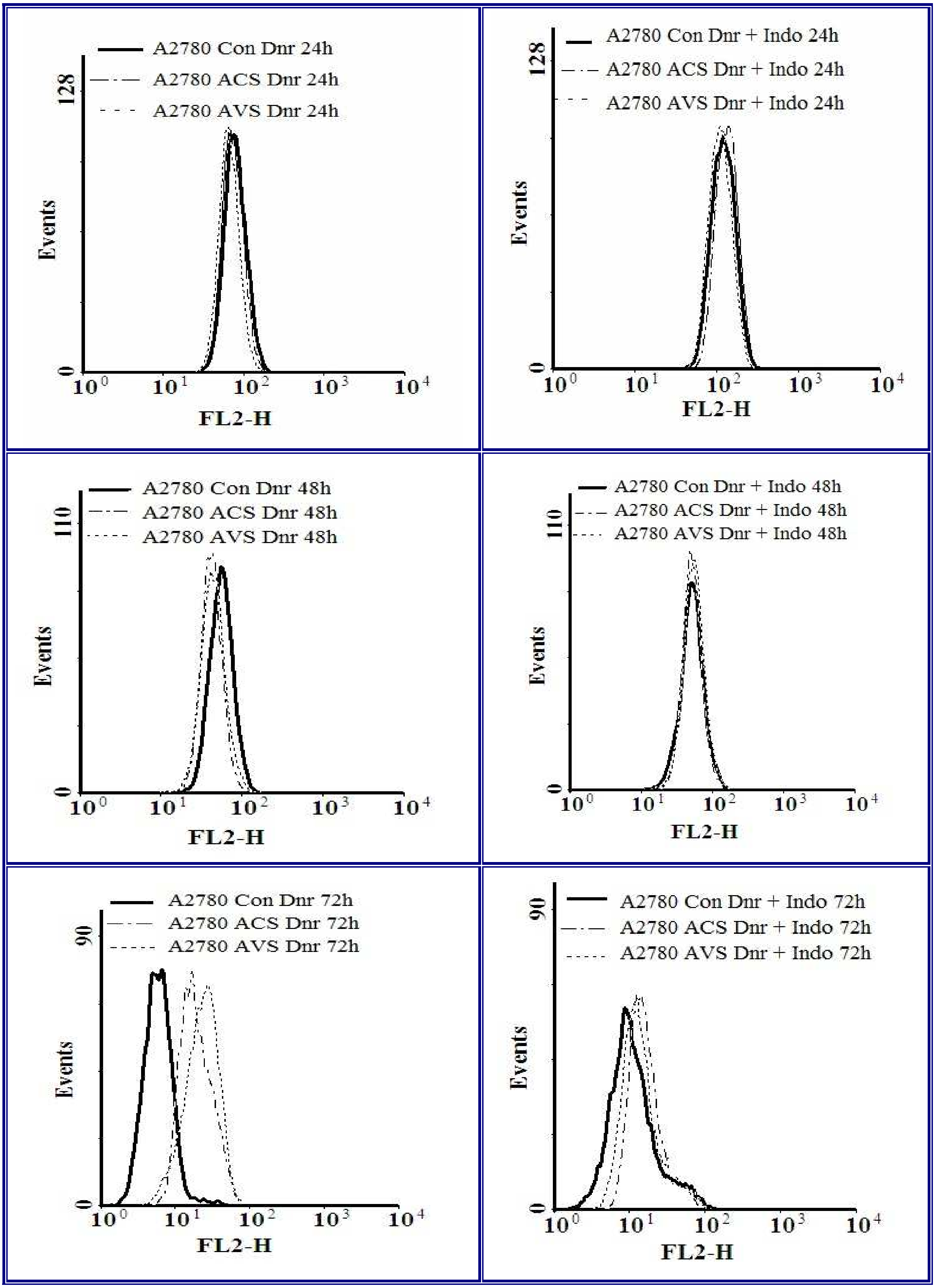
A2780 Con Dnr 24h
A2780 ACS Dnr 24h
A2780 AVS Dnr 24h
128
0
Events
10⁰ 10¹ 10² 10³ 10⁴
FL2-H
A2780 Con Dnr + Indo 24h
A2780 ACS Dnr + Indo 24h
A2780 AVS Dnr + Indo 24h
128
0
Events
10⁰ 10¹ 10² 10³ 10⁴
FL2-H
A2780 Con Dnr 48h
A2780 ACS Dnr 48h
A2780 AVS Dnr 48h
110
0
Events
10⁰ 10¹ 10² 10³ 10⁴
FL2-H
A2780 Con Dnr + Indo 48h
A2780 ACS Dnr + Indo 48h
A2780 AVS Dnr + Indo 48h
110
0
Events
10⁰ 10¹ 10² 10³ 10⁴
FL2-H
A2780 Con Dnr 72h
A2780 ACS Dnr 72h
A2780 AVS Dnr 72h
90
0
Events
10⁰ 10¹ 10² 10³ 10⁴
FL2-H
A2780 Con Dnr + Indo 72h
A2780 ACS Dnr + Indo 72h
A2780 AVS Dnr + Indo 72h
90
0
Events
10⁰ 10¹ 10² 10³ 10⁴
FL2-H

Supplementary Figure S4. Analyses of shikonin effects on daunorubicin and mitoxantrone efflux from EPG85.257, MCF7, and A2780 parent cells in the absence or presence of specific inhibitors (verapamil, novobiocin, or indomethacin). No drug (autofluorescence; solid line), fluorescence levels after 60 min of efflux in the presence of acetoxyisovalerylshikonin (dashed line) or acetylshikonin (dotted line). Con, control; AVS, acetoxyisovaleryl-shikonin; ACS, acetylshikonin; Dnr, daunorubicin; Indo, indomethacin; Mit, mitoxantrone; Novo, novobiocin; Ver, verapamil.

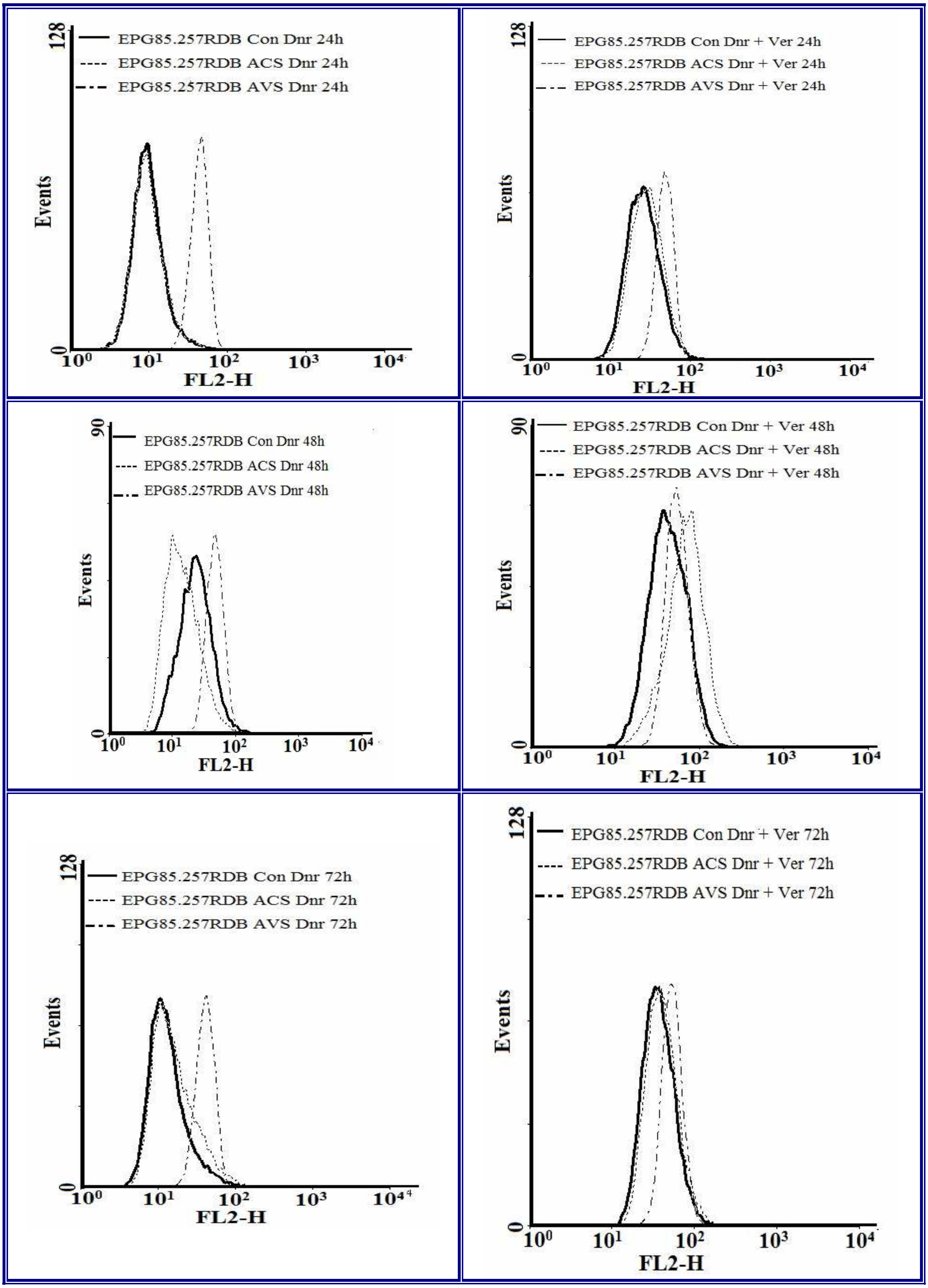
EPG85.257RDB Con Dnr 24h
EPG85.257RDB ACS Dnr 24h
EPG85.257RDB AVS Dnr 24h
EPG85.257RDB Con Dnr + Ver 24h
EPG85.257RDB ACS Dnr + Ver 24h
EPG85.257RDB AVS Dnr + Ver 24h
EPG85.257RDB Con Dnr 48h
EPG85.257RDB ACS Dnr 48h
EPG85.257RDB AVS Dnr 48h
EPG85.257RDB Con Dnr + Ver 48h
EPG85.257RDB ACS Dnr + Ver 48h
EPG85.257RDB AVS Dnr + Ver 48h
EPG85.257RDB Con Dnr 72h
EPG85.257RDB ACS Dnr 72h
EPG85.257RDB AVS Dnr 72h
EPG85.257RDB Con Dnr + Ver 72h
EPG85.257RDB ACS Dnr + Ver 72h
EPG85.257RDB AVS Dnr + Ver 72h
Events
FL2-H
128
90
0
10^0 10^1 10^2 10^3 10^4

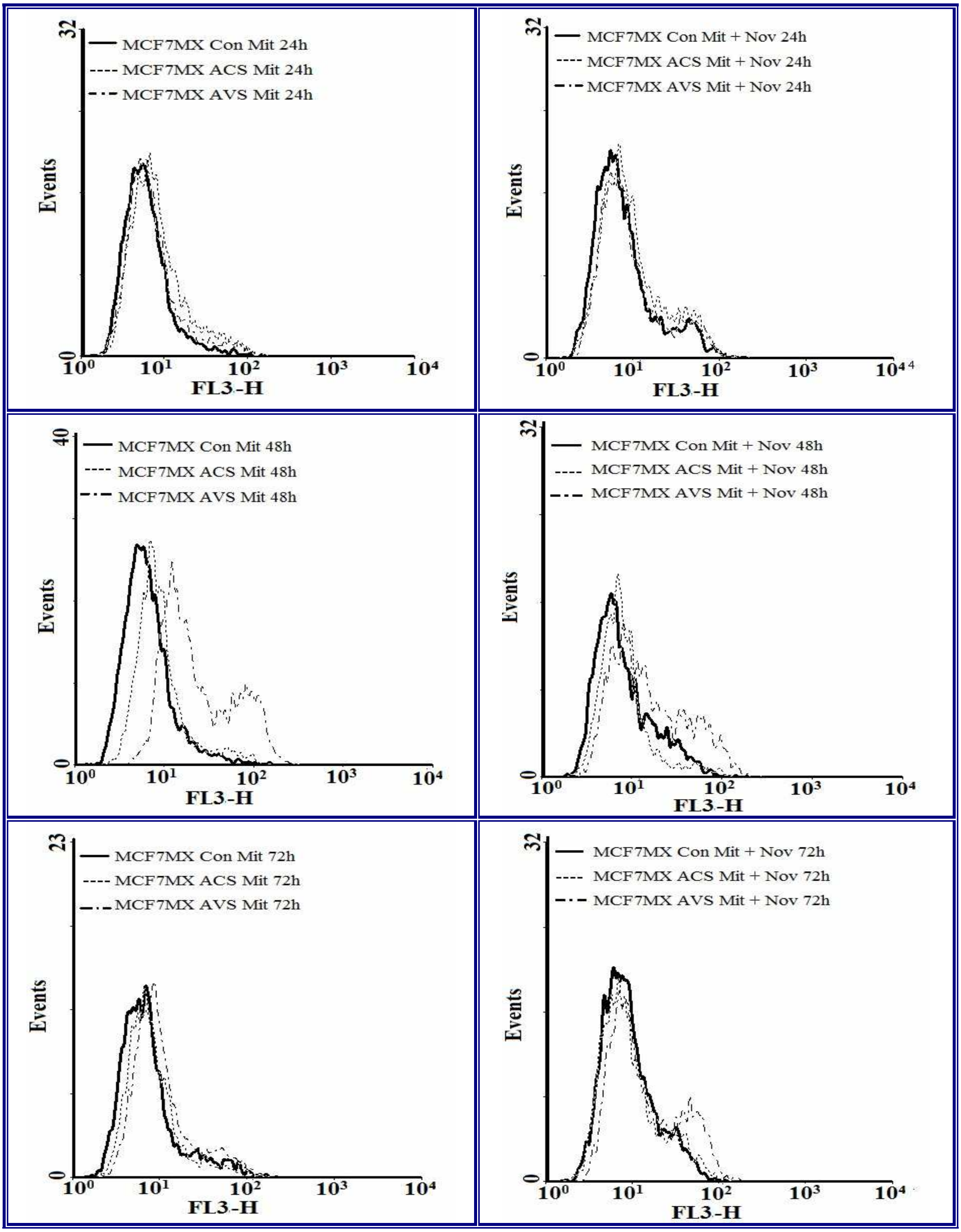
MCF7MX Con Mit 24h
MCF7MX ACS Mit 24h
MCF7MX AVS Mit 24h
Events
FL3-H
MCF7MX Con Mit + Nov 24h
MCF7MX ACS Mit + Nov 24h
MCF7MX AVS Mit + Nov 24h
MCF7MX Con Mit 48h
MCF7MX ACS Mit 48h
MCF7MX AVS Mit 48h
MCF7MX Con Mit + Nov 48h
MCF7MX ACS Mit + Nov 48h
MCF7MX AVS Mit + Nov 48h
MCF7MX Con Mit 72h
MCF7MX ACS Mit 72h
MCF7MX AVS Mit 72h
MCF7MX Con Mit + Nov 72h
MCF7MX ACS Mit + Nov 72h
MCF7MX AVS Mit + Nov 72h

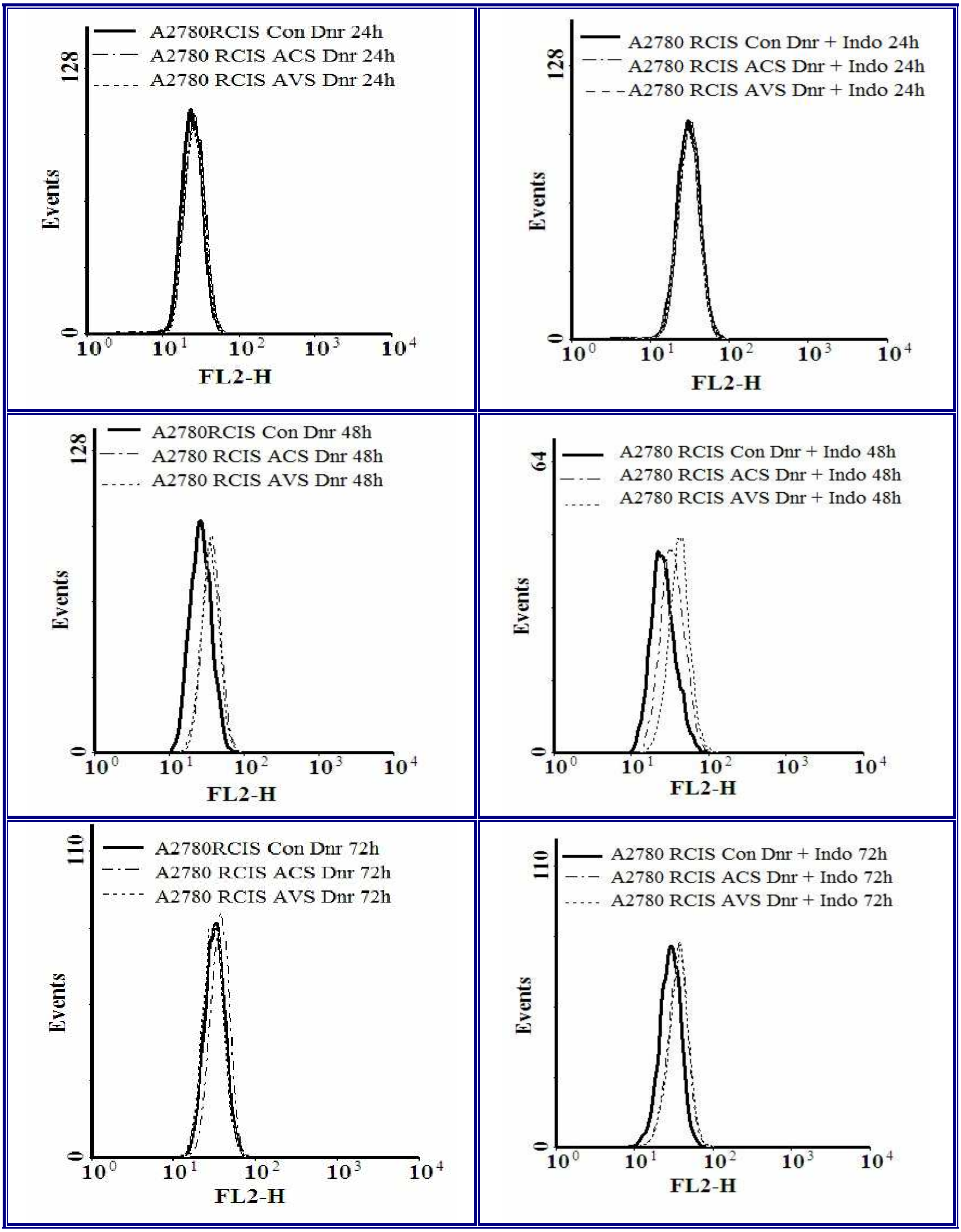

Supplementary Figure S5. Efflux analyses of shikonin derivatives on daunorubicin and mitoxantrone from EPG85.257RDB, MCF7MX, and A2780RCIS resistant cells in the absence or presence of specific inhibitors (verapamil, novobiocin, or

indomethacin). No drug (autofluorescence; solid line), fluorescence levels after 60 min of efflux in the presence of acetoxyisovalerylshikonin (dashed line) or acetylshikonin (dotted line). Right-shifted fluorescence peaks represent efflux inhibition. Con, control; AVS, acetoxyisovaleryl-shikonin; ACS, acetylshikonin; Dnr, daunorubicin; Indo, indomethacin; Mit, mitoxantrone; Novo, novobiocin; Ver, verapamil.

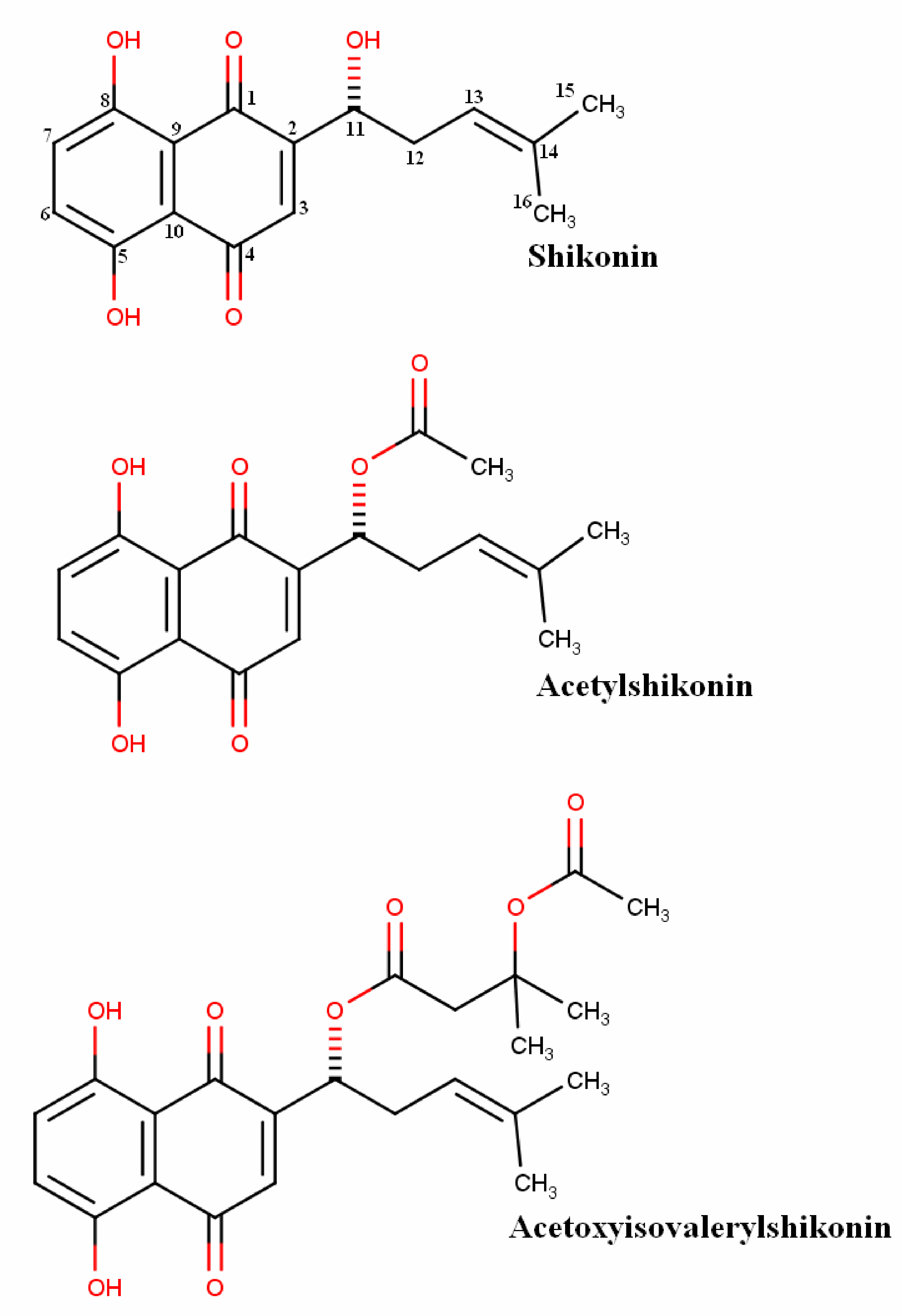

Supplementary figure S6. Chemical structures of shikonin, acetylshikonin, and acetoxyisovalerylshikonin.
